# Liquid and Tissue Biopsies for Identifying *MET* Exon 14 Skipping NSCLC: Analyses from the Phase II VISION Study of Tepotinib

**DOI:** 10.1158/1078-0432.CCR-24-4097

**Published:** 2025-05-01

**Authors:** Christian Rolfo, Aurora O’Brate, Christoph Menzel, Rolf Bruns, Dilafruz Juraeva, Christopher Stroh, Andreas Johne, Paul K. Paik

**Affiliations:** 1 Center for Thoracic Oncology, The Tisch Cancer Institute, Icahn School of Medicine at Mount Sinai, New York, New York.; 2 Global Medical Affairs, the healthcare business of Merck KGaA, Darmstadt, Germany.; 3 Global Companion Diagnostics, the healthcare business of Merck KGaA, Darmstadt, Germany.; 4 Department of Biostatistics, the healthcare business of Merck KGaA, Darmstadt, Germany.; 5 Oncology Data Science, the healthcare business of Merck KGaA, Darmstadt, Germany.; 6 Companion Diagnostics & Biomarker Strategy, the healthcare business of Merck KGaA, Darmstadt, Germany.; 7 Global Clinical Development, the healthcare business of Merck KGaA, Darmstadt, Germany.; 8 Department of Medicine, Thoracic Oncology Service, Memorial Sloan-Kettering Cancer Center, New York, New York.; 9 Department of Medicine, Weill Cornell Medical College, New York, New York.

## Abstract

**Purpose:**

The VISION trial of tepotinib, a selective MET inhibitor, enrolled patients with non–small cell lung cancer and prospectively detected *MET* exon 14 (*MET*ex14) skipping in liquid biopsies (LBx) and/or tissue biopsies (TBx). We evaluated patient characteristics and outcomes according to *MET*ex14 positivity in LBx (LBx-positive) or TBx (TBx-positive).

**Experimental Design:**

*MET*ex14 was centrally assessed by next-generation sequencing of ctDNA from LBx (Guardant360/ArcherMET) and/or RNA from TBx (Oncomine Focus/ArcherMET) or, in Japan only, local TBx PCR. Parallel LBx/TBx testing was recommended but not mandatory. Eligibility required LBx-positive or TBx-positive status. ctDNA burden was analyzed in patients with baseline Guardant360 data.

**Results:**

*MET*ex14 was detected in 469 of 7,937 prescreened/screened patients, 313 of whom were enrolled (TBx-positive, *n* = 208; LBx-positive, *n* = 178). LBx-positive patients had higher radiographic tumor burden than TBx-positive patients, including higher median sum of target lesion diameters per RECIST v1.1 (67.1 vs. 55.2 mm) and more patients with ≥3 target lesions (27.5% vs. 18.8%). In 180 TBx-positive patients with matching LBx results, objective response rates were slightly higher in TBx-positive/LBx-positive patients, but TBx-positive/LBx-negative patients had longer duration of response, progression-free survival, and overall survival. In ctDNA analysis (*n* = 165), detectable baseline ctDNA burden was associated with shorter progression-free survival and overall survival.

**Conclusions:**

Tepotinib had robust, durable activity in TBx-positive/LBx-negative and TBx-positive/LBx-positive patients. Although LBx is a complementary method to TBx for detecting *MET*ex14, it may preferentially select patients with higher tumor burden and poorer prognosis. Undetectable *MET*ex14 in baseline ctDNA (due to low ctDNA shedding) may define more favorable treatment outcomes.

**Translational Relevance**

The VISION trial of tepotinib is one of the first non–small cell lung cancer trials to enroll based on prospective detection of a driver alteration, namely, *MET* exon 14 skipping, in tissue biopsies (TBx) and/or liquid biopsies (LBx). Tepotinib showed robust, durable efficacy in TBx-positive and LBx-positive patients, with trends for longer time–dependent endpoints in the TBx-positive subgroup. Here, we report that LBx-positive patients had higher baseline disease burden, likely reflecting greater ctDNA shedding of larger tumors. In TBx-positive patients with matching LBx results, tepotinib exhibited durable efficacy in TBx-positive/LBx-positive and TBx-positive/LBx-negative patients, with more favorable outcomes associated with TBx-positive/LBx-negative status. These data underscore the suitability and complementarity of TBx and LBx for *MET* exon 14 skipping detection, while highlighting the potential of LBx to preferentially select patients with a poorer prognosis and higher tumor burden, which should be considered when interpreting trial data.

## Introduction

The management of advanced non–small cell lung cancer (NSCLC) has been revolutionized by the development of targeted therapies for tumors harboring driver alterations in *EGFR*, anaplastic lymphoma kinase, *MET*, and other genes (1). To support optimal treatment decisions, clinical practice guidelines recommend broad molecular profiling at diagnosis, for example, using targeted next-generation sequencing (NGS) panels to facilitate comprehensive interrogation of the wide range of clinically relevant markers (2, 3). Genetic material for NGS profiling is traditionally isolated from tumor tissue collected via transbronchial, percutaneous, or surgical biopsies (2). Although tissue biopsy (TBx) assays remain the diagnostic gold standard because of their ability to directly detect alterations in the tumor, they may not be feasible in patients with inaccessible tumors or poor clinical condition, preventing invasive biopsy collection (4, 5). Further challenges include delays due to biopsy procedure scheduling, inadequacy of sample quality and/or quantity, and sampling error caused by tumor heterogeneity (4, 5).

Many of these limitations may be overcome with liquid biopsies (LBx), i.e., blood samples, which can be analyzed by NGS of ctDNA shed by the tumor (4). Due to their minimally invasive nature, LBx are more patient-centric; they can be rapidly obtained from most patients, including those with poor clinical condition, and facilitate serial evaluation over time (4, 5). LBx may also surmount issues relating to tumor heterogeneity by collecting genetic material from the entire tumor (4, 5) and enable quantification of ctDNA burden as a potential surrogate for tumor burden (6). However, their sensitivity can be limited, especially in tumors with a low tumor fraction due to low ctDNA shedding or low variant allele fraction, leading to false-negative results (4, 5, 7, 8). LBx and TBx are therefore considered to play complementary clinical roles (4).

To inform the use of TBx and LBx in clinical practice, further evidence is needed to help understand differences in the patient populations identified by each method and their respective outcomes. Relevant data are provided by the phase II VISION trial of tepotinib, a highly selective, oral, once-daily MET tyrosine kinase inhibitor, in patients with advanced NSCLC with *MET* exon 14 (*MET*ex14) skipping (9–11), which is a predictive marker for MET inhibitors (12). As one of the first prospective trials to enroll patients based on detection of a biomarker (*MET*ex14 skipping) in TBx and/or LBx, VISION provides an important opportunity to investigate differences between patients identified with each biopsy type, who were otherwise selected and treated according to a common protocol.

Tepotinib demonstrated robust and durable clinical activity in the overall population, irrespective of prior treatment, including in preplanned subgroup analyses of patients with a TBx-positive or LBx-positive *MET*ex14 skipping result (11). Objective response rates (ORR) were similar between the TBx-positive and LBx-positive subgroups, at 54.3% and 51.7%, respectively. However, time-dependent endpoints showed trends for better outcomes in TBx-positive versus LBx-positive patients, with the median duration of response (DOR) of 18.0 and 15.2 months, median progression-free survival (PFS) of 13.7 and 8.9 months, and median overall survival (OS) of 22.9 and 17.6 months, respectively. To further evaluate differences in the populations identified by TBx and LBx in VISION, we report detailed prescreening data, baseline characteristics, and efficacy outcomes according to TBx and LBx status.

## Materials and Methods

### Study design

As reported previously (9–11), VISION was an international, single-arm, multicohort, open-label phase II trial (ClinicalTrials.gov; NCT02864992). The present analysis combines cohorts A (primary cohort) and C (confirmatory cohort) of patients with NSCLC with *MET*ex14 skipping.

Trial conduct was in accordance with the Declaration of Helsinki, International Conference on Harmonization Good Clinical Practice, local laws, and applicable regulatory requirements. Institutional review boards or independent ethics committees approved the protocol at each site. Patients provided written informed consent.

### Patients

Eligible patients had confirmed advanced NSCLC with *MET*ex14 skipping, measurable disease per RECIST v1.1, and Eastern Cooperative Oncology Group performance status 0–1. Patients could be treatment-naïve or have up to two prior lines of anticancer therapy.

*MET*ex14 skipping was centrally assessed in TBx and/or LBx during prescreening at any time prior to enrollment. Tumor tissue was derived from either archival samples collected at any time prior to enrollment or, if obtained with reasonable effort, freshly collected formalin-fixed, paraffin-embedded TBx. Blood samples for ctDNA isolation (i.e., LBx) were freshly collected in Cell-Free DNA BCT whole-blood collection tubes (Streck, Inc.) during prescreening. For patients with TBx, it was recommended that LBx were also obtained. Parallel testing of both TBx and LBx was highly recommended but not mandatory, and patients could enroll based on a positive result from one or both biopsy types. The average turnaround time for LBx- and TBx-based NGS testing was approximately 8 and 11 business days, respectively.

RNA isolated from TBx was analyzed using the Oncomine Focus Assay (52-gene panel; Thermo Fisher Scientific Inc.) or the ArcherMET assay (ArcherDX Inc.). ctDNA isolated from LBx was analyzed using the Guardant360 (73-gene panel; Guardant Health) or ArcherMET assays. Patients in Japan could enroll without prescreening via a nationwide cancer genomic screening project (LC-SCRUM) based on local detection of *MET*ex14 skipping in TBx using RT-PCR (13, 14).

### Study procedures and endpoints

Patients received once-daily oral tepotinib 500 mg (450 mg active moiety) until disease progression, unacceptable toxicity, or withdrawal for other reasons. The primary endpoint was objective response per RECIST v1.1, as assessed by an independent review committee. Secondary endpoints included DOR, PFS, OS, and health-related quality of life (HRQoL), assessed using the EQ-5D five-level version and European Organisation for Research and Treatment of Cancer (EORTC) Quality of Life Questionnaire-Core 30 (QLQ-C30) and Lung Cancer-13 questionnaires.

### Measures of disease burden and tumor heterogeneity

Baseline radiographic disease burden was described based on the sum of target lesion diameters (SOLD) and the number and sites of target and nontarget lesions, as evaluated by the independent review committee per RECIST v1.1. Baseline ctDNA burden was analyzed in *MET*ex14 skipping–positive (i.e., TBx-positive and/or LBx-positive) patients who had Guardant360 data. Silent mutations and variants without Catalogue of Somatic Mutations in Cancer identifiers were excluded from the analysis. Based on detection of any variant in any gene, ctDNA burden was categorized as “undetectable” (no variants detected) or “detectable” (≥1 variant detected). LBx-positive patients with Guardant360 data were also categorized according to the presence or absence of concomitant variants at baseline.

### Statistics

Analyses were conducted for all patients who received ≥1 dose of tepotinib. Preplanned analyses summarized baseline patient characteristics, including radiographic disease burden, overall and by treatment line, as well as HRQoL in TBx-positive and LBx-positive patients. Efficacy was also analyzed in TBx-positive patients with matched LBx results, according to LBx-positive or LBx-negative *MET*ex14 skipping status. Outcomes were summarized according to ctDNA burden (TBx-positive and/or LBx-positive patients) or the presence of concomitant alterations (LBx-positive patients) based on baseline Guardant360 data. Objective response was summarized as rates with two-sided Clopper–Pearson 95% confidence intervals (CI). Time-dependent endpoints were analyzed by Kaplan–Meier methods. All analyses were descriptive. The data cutoff was November 20, 2022.

### Data availability

Any requests for data by qualified scientific and medical researchers for legitimate research purposes will be subject to the healthcare business of Merck KGaA, Darmstadt, Germany’s (CrossRef Funder ID: 10.13039/100009945) Data Sharing Policy. All requests should be submitted in writing to the healthcare business of Merck KGaA, Darmstadt, Germany’s data sharing portal (https://www.emdgroup.com/en/research/our-approach-to-research-and-development/healthcare/clinical-trials/commitment-responsible-data-sharing.html). When the healthcare business of Merck KGaA, Darmstadt, Germany, has a co-research, co-development, or co-marketing or co-promotion agreement or when the product has been out-licensed, the responsibility for disclosure might be dependent on the agreement between parties. Under these circumstances, the healthcare business of Merck KGaA, Darmstadt, Germany, will endeavor to gain agreement to share data in response to requests.

## Results

### Patient disposition

**Figure 1.**
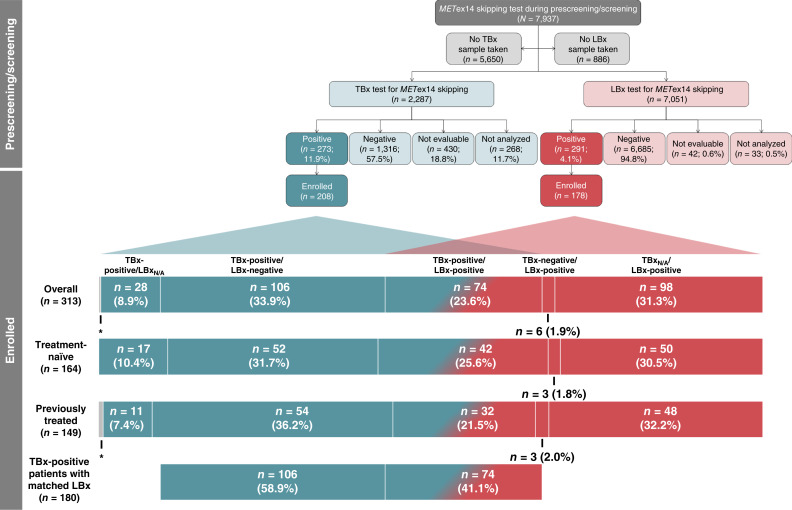
Patient prescreening/screening and enrollment. *One (0.3%) patient was enrolled based on local testing in a protocol violation. LBx-negative, negative for *MET*ex14 skipping in LBx; LBx-positive, positive for *MET*ex14 skipping in LBx; LBx_N/A_, no available result for *MET*ex14 skipping in LBx; TBx-negative, negative for *MET*ex14 skipping in TBx; TBx-positive, positive for *MET*ex14 skipping in TBx; TBx_N/A_, no available result for *MET*ex14 skipping in TBx.

TBx and/or LBx from 7,937 patients were submitted for *MET*ex14 skipping assessment during prescreening or screening between July 2016 and April 2021 (Fig. 1). A total of 2,266 patients provided TBx, which were analyzed using the Oncomine Focus Assay in 1,813 (80.0%) patients and the ArcherMET assay in 453 (20.0%) patients. A further 21 patients were evaluated by local tissue RT-PCR via the LC-SCRUM program in Japan. A total of 7,051 patients provided LBx, which were analyzed using the Guardant360 assay in 5,576 (79.1%) patients and the ArcherMET assay in 1,475 (20.9%) patients. Overall, 469 of 7,937 (5.9%) patients had a positive *MET*ex14 skipping result from TBx and/or LBx during prescreening/screening. Among 1,032 patients with both TBx and LBx results, 225 (21.8%) were TBx-positive and 105 (10.2%) were LBx-positive.

A total of 208 of 273 (76.2%) TBx-positive patients and 178 of 291 (61.2%) LBx-positive patients were enrolled. The total study population comprised 313 patients, of whom 164 were treatment-naïve and 149 were previously treated.

### Patient characteristics according to TBx and LBx status

Baseline demographics were broadly comparable between TBx-positive (*n* = 208) and LBx-positive patients (*n* = 178) overall and in the treatment-naïve and previously treated subgroups (Table 1). However, a lower proportion of TBx-positive versus LBx-positive patients were White (57.7% vs. 69.1%) or were enrolled in Europe (42.3% vs. 52.2%). These differences were more pronounced in treatment-naïve patients (White: 61.3% vs. 76.8%, respectively; enrolled in Europe: 44.1% vs. 56.8%). In treatment-naïve patients, TBx-positive patients were less likely than LBx-positive patients to be enrolled in North America (14.4% vs. 20.0%) or have Eastern Cooperative Oncology Group performance status 1 (70.3% vs. 75.8%).

**Table 1.** Baseline characteristics of TBx-positive and LBx-positive patients, overall and according to prior treatment.

Characteristic	Overall (*n* = 313)		Treatment-naïve (*n* = 164)		Previously treated (*n* = 149)	
	TBx-positive (*n* = 208)	LBx-positive (*n* = 178)	TBx-positive (*n* = 111)	LBx-positiv (*n* = 95)	TBx-positive (*n* = 97)	LBx-positive (*n* = 83)
Median age, years (range)	72.7 (41–94)	71.2 (47–89)	75.0 (47–94)	71.6 (47–88)	70.3 (41–89)	70.8 (49–89)
Age group, *n* (%)						
<65 years	40 (19.2)	41 (23.0)	17 (15.3)	21 (22.1)	23 (23.7)	20 (24.1)
65 to <75 years	84 (40.4)	70 (39.3)	39 (35.1)	38 (40.0)	45 (46.4)	32 (38.6)
75 to <85 years	67 (32.2)	56 (31.5)	44 (39.6)	28 (29.5)	23 (23.7)	28 (33.7)
≥85 years	17 (8.2)	11 (6.2)	11 (9.9)	8 (8.4)	6 (6.2)	3 (3.6)
Sex, *n* (%)						
Male	108 (51.9)	83 (46.6)	59 (53.2)	46 (48.4)	49 (50.5)	37 (44.6)
Female	100 (48.1)	95 (53.4)	52 (46.8)	49 (51.6)	48 (49.5)	46 (55.4)
Race, *n* (%)						
White	120 (57.7)	123 (69.1)	68 (61.3)	73 (76.8)	52 (53.6)	50 (60.2)
Asian	83 (39.9)	48 (27.0)	42 (37.8)	21 (22.1)	41 (42.3)	27 (32.5)
Other^a^	2 (1.0)	2 (1.1)	0	1 (1.1)	2 (2.1)	1 (1.2)
Geographic region, *n* (%)						
North America	33 (15.9)	37 (20.8)	16 (14.4)	19 (20.0)	17 (17.5)	18 (21.7)
Europe	88 (42.3)	93 (52.2)	49 (44.1)	54 (56.8)	39 (40.2)	39 (47.0)
Asia	87 (41.8)	48 (27.0)	46 (41.4)	22 (23.2)	41 (42.3)	26 (31.3)
Smoking history, *n* (%)^b^						
Yes	98 (47.1)	84 (47.2)	58 (52.3)	50 (52.6)	40 (41.2)	34 (41.0)
No	100 (48.1)	90 (50.6)	52 (46.8)	45 (47.4)	48 (49.5)	45 (54.2)
ECOG PS, *n* (%)^c^						
0	57 (27.4)	42 (23.6)	32 (28.8)	23 (24.2)	25 (25.8)	19 (22.9)
1	150 (72.1)	136 (76.4)	78 (70.3)	72 (75.8)	72 (74.2)	64 (77.1)
Histology, *n* (%)^d^						
Adenocarcinoma	170 (81.7)	143 (80.3)	90 (81.1)	77 (81.1)	80 (82.5)	66 (79.5)
Squamous	14 (6.7)	20 (11.2)	6 (5.4)	9 (9.5)	8 (8.2)	11 (13.3)
Sarcomatoid	5 (2.4)	6 (3.4)	3 (2.7)	5 (5.3)	2 (2.1)	1 (1.2)
Other	17 (8.2)	9 (5.1)	11 (9.9)	4 (4.2)	6 (6.2)	5 (6.0)
Median SOLD per RECIST v1.1, mm (range)	55.2 (10.2–267.5)	67.1 (11.6–227.8)	58.1 (10.2–267.5)	66.4 (12.3–227.8)	52.7 (11.6–178.3)	69.2 (11.6–178.3)

Abbreviations: ECOG PS, Eastern Cooperative Oncology Group performance status; LBx-positive, positive for *MET*ex14 skipping in LBx; TBx-positive, positive for *MET*ex14 skipping in TBx.

a Race was Black or African American in three patients, “other” in one patient, and missing in eight patients.

b Smoking history was missing in 10 patients.

c ECOG PS was 2 in one patient.

d Histology was missing in two patients.

**Figure 2.**
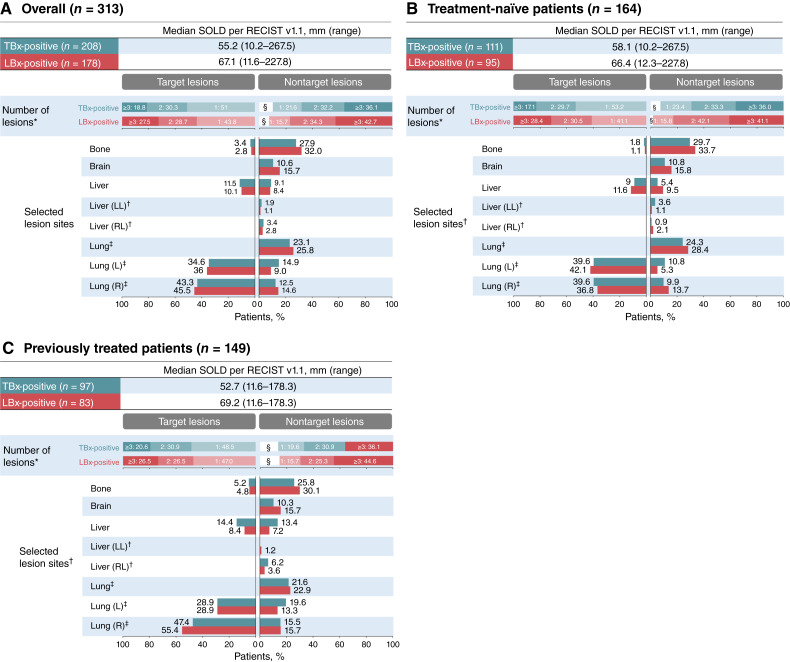
Baseline radiographic disease burden highlighting SOLD per RECIST v1.1; the proportion of patients with ≥3 target lesions and selected lesion sites in (**A**) the overall population, (**B**) treatment-naïve patients, and (**C**) previously treated patients.*Target and nontarget lesions by an independent review committee. †“Liver (LL)” and “liver (RL)” categories were not included for target lesions. ^‡^Lung lesion site was categorized as “lung”, “lung (L)”, or “lung (R)” for target and nontarget lesions, but no target lesions were reported in the overall “lung” category. ^§^No nontarget lesions were reported for 1.1% of treatment-naïve LBx-positive patients. L, left; LBx-positive, positive for *MET*ex14 skipping in LBx; LL, left lobe; R, right; RL, right lobe; TBx-positive, positive for *MET*ex14 skipping in TBx.

Baseline clinical characteristics indicated lower radiographic disease burden in TBx-positive than in LBx-positive patients, with the median (range) SOLD per RECIST v1.1 of 55.2 mm (10.2–267.5) and 67.1 mm (11.6–227.8), respectively (Fig. 2A; Table 1). In addition, lower proportions of TBx-positive versus LBx-positive patients had ≥3 target lesions (18.8% vs. 27.5%), ≥3 nontarget lesions (36.1% vs. 42.7%), or nontarget lesions in the brain (10.6% vs. 15.7%) or bone (27.9% vs. 32.0%). These differences were evident in both treatment-naïve and previously treated patients (Fig. 2B and C; Table 1).

TBx-positive patients had better overall HRQoL than LBx-positive patients at baseline, as reflected in the mean (± SD) EQ-5D five-level visual analog scale score (67 ± 19.0 vs. 63 ± 20.8) and EORTC QLQ-C30 global health scores (60.1 ± 22.5 vs. 53.9 ± 24.1; Table 2). Of the EORTC QLQ-C30 patient functioning scales, the greatest difference was seen for role functioning. EORTC Quality of Life Questionnaire-Lung Cancer-13 symptom scores suggested lower severity of cough and dyspnea in TBx-positive than LBx-positive patients.

**Table 2.** Baseline HRQoL in TBx-positive and LBx-positive patients.

Mean (SD)	TBx-positive (*n* = 208)	LBx-positive (*n* = 178)
EORTC QLQ-LC13 symptom scores^a^		
Cough	30.7 (27.3)	34.2 (29.6)
Dyspnea	24.9 (20.1)	29.0 (24.1)
Chest pain	20.1 (28.1)	19.0 (26.4)
EORTC QLQ-C30 patient functioning scales^b^		
Global health score	60.1 (22.5)	53.9 (24.1)
Physical	72.1 (23.0)	69.0 (25.6)
Role	71.7 (30.0)	65.3 (32.1)
Emotional	75.7 (22.8)	72.3 (23.6)
Cognitive	82.1 (20.6)	81.5 (22.2)
Social	76.9 (25.6)	72.4 (29.5)
EQ-5D-5L^b^		
VAS	67 (19.0)	63 (20.8)

Abbreviations: EORTC QLQ-LC13, European Organisation for Research and Treatment of Cancer-Lung Cancer-13; EQ-5D-5L, EQ-5D five-level version; LBx-positive, positive for *MET*ex14 skipping in LBx; TBx-positive, positive for *MET*ex14 skipping in TBx; VAS, visual analog scale.

a Lower scores indicate milder symptoms.

b Higher scores indicate greater function.

### Efficacy in TBx-positive patients with matched LBx results

Of 313 patients in the study, 186 were enrolled with paired TBx and LBx results; 74 (23.6%) patients were TBx-positive/LBx-positive, 106 (33.9%) were TBx-positive/LBx-negative, and 6 (1.9%) were TBx-negative/LBx-positive. A further 98 (31.3%) patients were LBx-positive with no available TBx result, and 28 (8.9%) patients were TBx-positive with no available LBx result (either due to unavailability of biopsy material or test failure). One (0.3%) patient was enrolled based on local testing in a protocol violation.

A total of 180 of 208 (86.5%) TBx-positive patients had matched LBx results, including 94 of 111 (84.7%) treatment-naïve patients and 86 of 97 (88.7%) previously treated patients. In treatment-naïve patients, *MET*ex14 skipping was undetectable in ctDNA (i.e., TBx-positive/LBx-negative) in 52 of 94 (55.3%) and detectable (TBx-positive/LBx-positive) in 42 of 94 (44.7%) patients. Among previously treated patients, 54 of 86 (62.8%) were TBx-positive/LBx-negative, and 32 of 86 (37.2%) were TBx-positive/LBx-positive for *MET*ex14 skipping.

**Figure 3.**
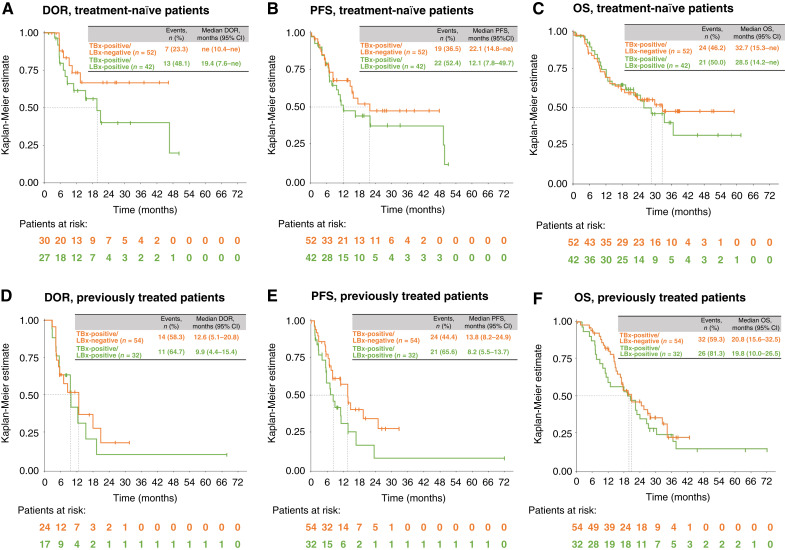
DOR* (**A** and **D**), PFS (**B** and **E**), and OS (**C** and **F**) in TBx-positive patients with matched LBx results for the treatment-naïve (**A–C**) and previously treated (**D–F**) subgroups. *Only patients with a response were included in Kaplan–Meier analyses of DOR. LBx-negative, negative for *MET*ex14 skipping in LBx; LBx-positive, positive for *MET*ex14 skipping in LBx; TBx-positive, positive for *MET*ex14 skipping in TBx.

Overall, meaningful durable efficacy was observed in treatment-naïve and previously treated TBx-positive patients, irrespective of the LBx result. In the treatment-naïve subgroup, TBx-positive/LBx-negative patients had slightly lower ORR (57.7; 95% CI, 43.2–71.3) than TBx-positive/LBx-positive patients (64.3%; 95% CI, 48.0–78.4). However, there were trends for longer time–dependent endpoints in TBx-positive/LBx-negative versus TBx-positive/LBx-positive patients (Fig. 3A–C). The median DOR was not estimable (ne; 95% CI, 10.4–ne) versus 19.4 months (95% CI, 7.6–ne), the median PFS was 22.1 (95% CI, 14.8–ne) versus 12.1 months (95% CI, 7.8–49.7), and the median OS was 32.7 (95% CI, 15.3–ne) versus 28.5 months (95% CI, 14.2–ne), respectively.

Similar trends were seen in previously treated patients, with ORRs of 44.4% (95% CI, 30.9–58.6) in TBx-positive/LBx-negative patients and 53.1% (95% CI, 34.7–70.9) in TBx-positive/LBx-positive patients. The median DOR was 12.6 (95% CI, 5.1–20.8) versus 9.9 (95% CI, 4.4–15.4) months, the median PFS was 13.8 (95% CI, 8.2–24.9) versus 8.2 (95% CI, 5.5–13.7) months, and the median OS was 20.8 (95% CI, 15.6–32.5) versus 19.8 (95% CI, 10.0–26.5) months, respectively (Fig. 3D–F).

### ctDNA analyses

**Figure 4.**
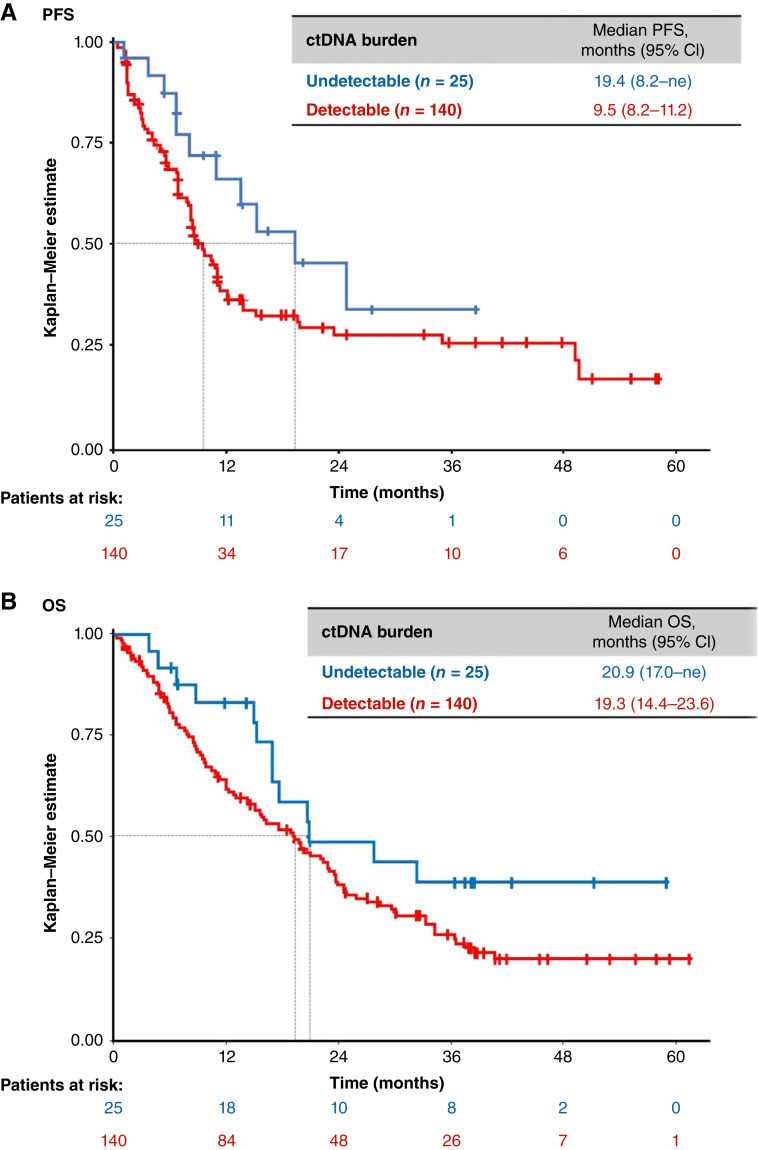
PFS (**A**) and OS (**B**) according to ctDNA burden in patients with baseline Guardant360 LBx data (*n* = 165).

Baseline Guardant360 LBx data were available for 165 patients, of whom 88 (53.3%) were TBx-positive and 114 (69.1%) were LBx-positive for *MET*ex14 skipping. Based on detection of any variant in any gene, 140 (84.8%) had detectable ctDNA and 25 (15.2%) had undetectable ctDNA. A higher proportion of patients in the detectable versus undetectable ctDNA group had ≥3 target lesions (25.7% vs. 4.0%), ≥3 nontarget lesions (45.0% vs. 16.0%), or ≥3 target or nontarget lesions (82.9% vs. 60.0%) per RECIST v1.1. The median PFS was shorter in patients with detectable ctDNA (9.5 months; 95% CI, 8.2–11.2) than in patients with undetectable ctDNA (19.4 months; 95% CI, 8.2–ne; Fig. 4A). There was also a trend for shorter OS in patients with detectable versus undetectable ctDNA, with the median OS of 19.3 (95% CI, 14.4–23.6) and 20.9 (95% CI, 17.0–ne) months, respectively (Fig. 4B).

In 114 LBx-positive patients, outcomes were better in patients without concomitant alterations detected in ctDNA (*n* = 38) than in patients with concomitant alterations (*n* = 76). The ORR (95% CI) was 65.8% (48.6–80.4) versus 43.4% (32.1–55.3), the median DOR (95% CI) was 19.4 (9.0–ne) versus 9.7 (6.6–33.6) months, the median PFS (95% CI) was 13.8 (8.3–49.7) versus 6.9 (5.5–9.6) months, and the median OS (95% CI) was 24.6 (17.6–38.6) versus 14.4 (9.5–19.8) months, respectively (Supplementary Fig. S1).

### Biopsy timing

In 187 TBx-positive patients with available tissue sampling dates, the median time from TBx collection to tepotinib initiation was 85 days (IQR, 43–306; range, 13–2,838). Among these 187 patients, the median time from TBx collection to tepotinib initiation was 64 days (IQR, 42–106; range, 13–1,774) for the 105 treatment-naïve patients and 168 days (IQR, 61–483; range, 15–2,838) for the 82 previously treated patients. TBx were collected before LBx in 61 TBx-positive/LBx-positive patients, with a median duration between the two biopsies of 44 days (IQR, 22–190; range, 8–1,111).

## Discussion

The VISION trial established the robust and durable long-term efficacy of tepotinib across treatment lines in patients with NSCLC and *MET*ex14 skipping in TBx and/or LBx (9–11). Although clinically meaningful efficacy was observed irrespective of biopsy type, there were trends for longer DOR, PFS, and OS in TBx-positive versus LBx-positive patients (11). The present analysis extends these findings by reporting durable efficacy of tepotinib in TBx-positive patients with undetectable or detectable *MET*ex14 skipping in ctDNA at baseline, as prospectively assessed in the largest population of patients with matched samples in an MET inhibitor trial. The ORR was slightly lower in TBx-positive/LBx-negative versus TBx-positive/LBx-positive patients, but TBx-positive/LBx-negative patients showed trends for longer time–dependent endpoints in both the treatment-naïve and previously treated subgroups. The efficacy of tepotinib was especially notable in treatment-naïve TBx-positive/LBx-negative patients, in whom the median DOR was not reached, the median PFS was 22.1 months, and the median OS was 32.7 months. Therefore, in TBx-positive patients, undetectable *MET*ex14 skipping in LBx (likely reflecting lower ctDNA burden) at baseline may define a more favorable treatment outcome.

Other MET inhibitor trials in *MET*ex14 skipping NSCLC enrolled only TBx-positive patients and retrospectively analyzed *MET*ex14 skipping in baseline ctDNA samples (15–17). Consistent with the trends in VISION, TBx-positive/LBx-negative patients (*n* = 20) versus TBx-positive/LBx-positive (*n* = 46) patients in the phase II savolitinib trial had longer median PFS (13.8 vs. 5.6 months) and median OS (ne vs. 10.9 months), but lower ORR (30.0% vs. 52.2%), in the overall population of treatment-naïve and previously treated patients (15). TBx-positive/LBx-negative patients also had significantly lower median SOLD per RECIST v1.1 (51.8 vs. 95.6 mm) and blood-based tumor mutational burden (1.06 vs. 5.29) than TBx-positive/LBx-positive patients (both *P*<0.001). In the PROFILE 1001 trial, the median PFS with crizotinib was longer in TBx-positive/LBx-negative (*n* = 19) versus TBx-positive/LBx-positive (*n* = 18) patients (8.1 vs. 3.7 months), but the ORR was comparable (24% vs. 19%; ref. 17). The GEOMETRY mono-1 trial of capmatinib reported outcomes for TBx-positive/LBx-positive patients but not for the TBx-positive/LBx-negative population (16). In treatment-naïve cohort 5b, 16 patients were TBx-positive/LBx-positive and 10 were TBx-positive/LBx-negative, whereas in previously treated cohort 4, 41 patients were TBx-positive/LBx-positive and 16 were TBx-positive/LBx-negative. The ORR was greater in the TBx-positive/LBx-positive subgroup than in the overall population (including both TBx-positive/LBx-positive and TBx-positive/LBx-negative patients), but time-dependent endpoints were mostly similar.

In VISION, LBx-positive patients had higher median SOLD per RECIST v1.1 than TBx-positive patients, with more patients with ≥3 target or nontarget lesions and a higher incidence of brain or bone metastases. The higher disease burden in LBx-positive patients is consistent with the reported correlation between tumor burden and ctDNA levels (18) and may account for their shorter time–dependent endpoints. In support of this explanation, detectable baseline ctDNA burden, which may be a surrogate for overall disease burden (6), was associated with shorter PFS and OS. Prior studies have demonstrated independent negative prognostic significance of both radiographic tumor size metrics (19–21) and ctDNA burden (22) in NSCLC. The greater tumor burden in LBx-positive patients may underlie their worse baseline performance status and HRQoL, which are themselves poor prognostic factors (23, 24). Our observation of similar ORR but shorter time–dependent endpoints in LBx-positive versus TBx-positive patients is consistent with a recent pan-cancer meta-analysis showing that higher radiographic burden independently predicted lower PFS and OS, but not ORR, in patients receiving targeted therapies (25).

The higher tumor burden in the LBx-positive subgroup likely reflects the reduced possibility of detecting alterations in LBx in patients with tumors with low ctDNA shedding (leading to a low tumor fraction), which is associated with smaller tumors and less advanced metastatic stage (i.e., M1a; refs. 4, 8, 26). Whereas ctDNA may remain below the limit of detection in patients with smaller tumors, patients with larger or more advanced metastatic tumors may be preferentially identified because of higher ctDNA shedding (27). ctDNA levels are also associated with higher metabolic activity and aggressive growth (28).

Collectively, our data imply that the use of LBx rather than TBx for trial enrollment may lead to less favorable efficacy outcomes because of preferential selection of patients with higher tumor burden and therefore a poorer prognosis. Differences in populations identified by TBx and LBx should be considered when interpreting trial data. Nonetheless, a major advantage of LBx for patient selection is its wide applicability, which enables molecular profiling even in patients with poor clinical condition, inaccessible tumors, or limited TBx quality or quantity (4, 5). Consistent with its expansion of the evaluable patient pool, incorporation of LBx testing for VISION trial eligibility substantially boosted enrollment (29), and almost one third of the population was enrolled based solely on LBx. Such patients would not have been enrolled in other MET inhibitor trials, which used only TBx to determine eligibility (17, 30, 31). The ease of biopsy is particularly relevant for patients with *MET*ex14 skipping NSCLC, who are underrepresented in therapeutic trials, partly because of their older age, and in whom TBx can be harder to obtain. Indeed, tepotinib is the only MET inhibitor that has demonstrated efficacy in a population of LBx-positive patients, including patients with unavailable TBx, in a prospective clinical trial. LBx also avoid adverse events of TBx procedures. Although VISION did not capture biopsy adverse events, which would have occurred before informed consent, major complication rates have been reported elsewhere at 13.4% for bronchoscopy, 11.3% for surgery, and 4.0% for cytology/needle biopsy (32). Patients with *MET*ex14 skipping may be at an elevated risk of pneumothorax or other complications due to their advanced age (12, 33, 34). LBx may also enable more comprehensive evaluation of tumor heterogeneity that is less susceptible to sampling variability than TBx, as well as serial tracking of clonal evolution over time. In LBx-positive patients in VISION, concomitant alterations in baseline ctDNA (employed as a simple measure of tumor heterogeneity) were associated with substantially poorer outcomes, consistent with a role for tumor heterogeneity as a key determinant of treatment resistance and tumor progression (35). Finally, LBx can be applied for longitudinal monitoring of molecular response, as illustrated by on-treatment data from VISION showing improved clinical outcomes in patients with confirmed molecular response or sustained undetectable *MET*ex14 skipping in ctDNA (36).

Prior data indicate that actionable genetic drivers generally remain detectable over the course of treatment for metastatic cancers (37). Accordingly, in TBx-positive patients, *MET*ex14 skipping was detected in LBx collected up to 1,111 days after the positive TBx, supporting the stability of *MET*ex14 skipping over time.

Collectively, the VISION trial data indicate that TBx and LBx are both suitable for the detection of *MET*ex14 skipping and should ideally be used together as complimentary methods at diagnosis to maximize the identification of candidates for MET inhibitor treatment. Consistent with the “tissue-first” and “plasma-first” approaches recommended by the International Association for the Study of Lung Cancer (4), as well as the tepotinib US Prescribing Information (38), a positive result from either biopsy type can be considered sufficient for MET inhibitor eligibility. A negative LBx result may be followed up by tissue testing, where possible, to minimize missed opportunities to receive an MET inhibitor due to false-negative results caused by low ctDNA shedding. Recent data suggest that reflex tissue testing is especially important in patients with low ctDNA levels (tumor fraction <1%), who have an appreciable risk of false-negative results, but may add limited value in patients with high ctDNA levels (tumor fraction ≥1%; ref. 8). Other potential sources of assay discordance include technical variation and tumor heterogeneity (causing false-negative TBx results; ref. 39).

Although TBx and LBx can be used as complimentary methods at diagnosis, the use of two distinct assays may constitute a potential limitation of the study. The serial profiling of ctDNA can complement baseline profiling to identify clinically actionable acquired genomic alterations prior to RECIST-defined progression and to understand their impact on patient prognosis, but this was not a focus of this study and has been reported elsewhere (36). Other limitations of the present analysis include its descriptive nature, which did not incorporate the formal statistical comparison of outcomes between biopsy subgroups. Measures of radiographic disease burden may have been systematically biased by the RECIST rules for lesion designation, whereas the utility of ctDNA as a surrogate for tumor burden may be limited by its association with proliferative rate, tumor site, vascularity, or other variables (28, 40).

In conclusion, in VISION, the only trial in *MET*ex14 skipping NSCLC to enroll based on LBx and/or TBx, tepotinib demonstrated robust and durable clinical activity in TBx-positive patients with LBx-negative or LBx-positive status. Although LBx are a complementary method to TBx for detecting *MET*ex14 skipping, they may preferentially select patients with a poorer prognosis and higher tumor burden, whereas patients with undetectable *MET*ex14 skipping in baseline ctDNA (reflecting lower ctDNA burden) may have a more favorable outcome. LBx may be a valuable tool for clinical trial enrollment that may be employed to complement tissue testing or as an initial testing strategy using a “plasma-first” approach.

## Supplementary Material

Supplementary Figure S1 Figure S1. DOR (A), PFS (B), and OS (C) according to the presence or absence of concomitant alterations in ctDNA in LBx-positive patients by Guardant360 (n = 114). CI, confidence interval; ctDNA, circulating tumor DNA; DOR, duration of response; OS, overall survival; PFS, progression-free survival.
