# Supplementary material for: Liquid and Tissue Biopsies for Identifying MET Exon 14 Skipping NSCLC: Analyses from the Phase II VISION Study of Tepotinib
Source: Clin Cancer Res. 2025 May 1;31(13):2675–84. doi: 10.1158/1078-0432.CCR-24-4097 (PMC12209826; doi:10.1158/1078-0432.CCR-24-4097)

**Figure S1.** DOR (A), PFS (B), and OS (C) according to the presence or absence of concomitant alterations in ctDNA in LBx-positive patients by Guardant360^®^ (n=114). CI, confidence interval; ctDNA, circulating tumor DNA; DOR, duration of response; OS, overall survival; PFS, progression-free survival.


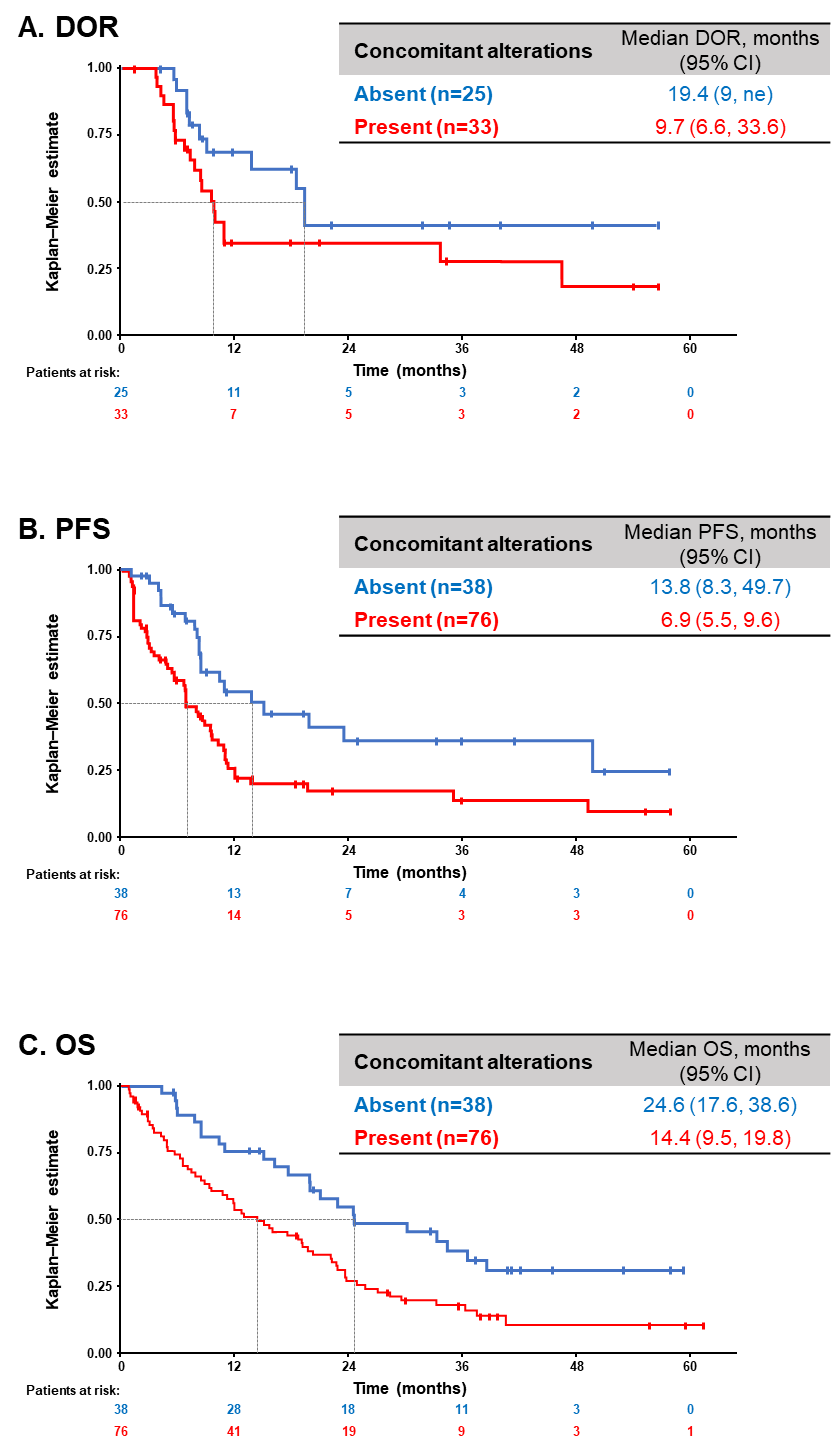

Supplement: Supplementary Figure S1 — Figure S1. DOR (A), PFS (B), and OS (C) according to the presence or absence of concomitant alterations in ctDNA in LBx-positive patients by Guardant360 (n = 114). CI, confidence interval; ctDNA, circulating tumor DNA; DOR, duration of response; OS, overall survival; PFS, progression-free survival. [file ccr-24-4097_supplementary_figure_s1_suppfs1.docx]
